# Gangliocytic Paraganglioma of Duodenum

**DOI:** 10.1155/2013/378582

**Published:** 2013-08-29

**Authors:** Vikram Narang, Nitin Behl, Neena Sood, Harpreet Puri

**Affiliations:** ^1^Department of Pathology, Dayanand Medical College Hospital, Ludhiana 141001, India; ^2^Department of Gastroenterology, Dayanand Medical College Hospital, Ludhiana 141001, India

## Abstract

Gangliocytic paragangliomas are rare benign tumors which are usually encountered in the second portion of the duodenum. Histogenesis of these tumors is incompletely understood. Patients usually present with upper gastrointestinal bleeding. The endoscopic features of gangliocytic paraganglioma do not differ from those of other submucosal tumors. Therefore, they can be diagnosed histologically by the presence of epithelioid, spindle, and ganglion cells, which is similar to that observed for paraganglioma. We herein report a case of gangliocytic paraganglioma due to the rarity of the lesion and the characteristic histopathologic findings.

## 1. Introduction

Gangliocytic paraganglioma derived from the neural crest is a peculiar neuroendocrine tumor, and 88% of the reported lesions are located in the second part of the duodenum [1]. Gangliocytic paraganglioma is characterized by a benign behavior and a favorable outcome. Localexcision is used to treat the disease, and radical surgery and lymph node dissection can be avoided if gangliocytic paraganglioma is confirmed. Here, we report a 32-year-old man with gangliocytic paraganglioma of the duodenum [2]. Local resection was performed.

## 2. Case Report

**Figure 1:**
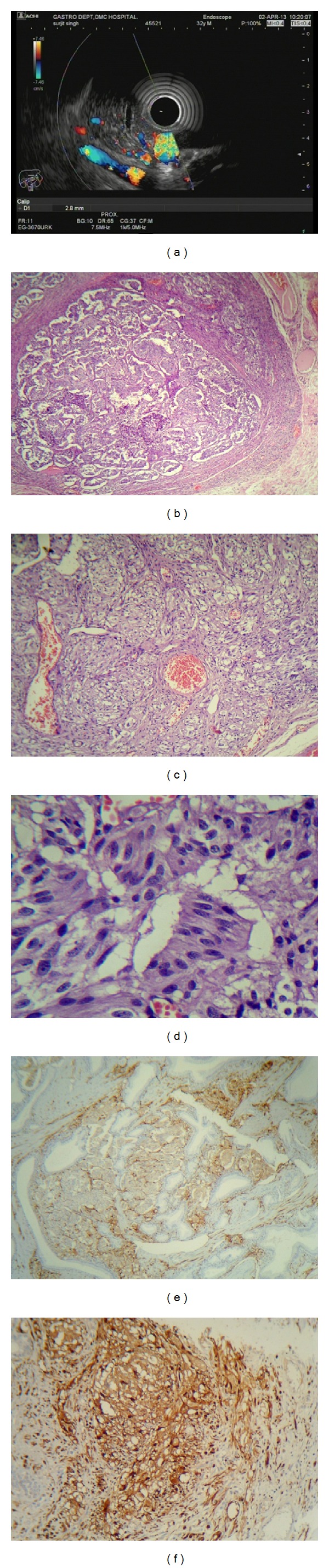
(a) Ultrasound color Doppler highlighting the submucosal lesion with increased vascularity in the second part of the duodenum. (b) Circumscribed lesion in submucosa, with tumor arranged in nesting pattern (H&E 100x). (c) Tumor cells showing abundant cytoplasm and mildly pleomorphic nuclei-ganglion cell morphology (H&E 400x). (d) Spindle cell morphology of tumor cells (H&E 40x). (e) Tumor cells showing synaptophysin positivity. (f) Tumor cells showing strong reaction with S-100 immunohistochemical stain.

A 32-year-old male presented to the gastroenterology outpatient department with complaints of mild epigastric discomfort and malena for about one week. History taking revealed no particular issues other than being occasionally alcoholic. Esophagogastroduodenoscopy revealed a submucosal vascular lesion measuring 2.3 × 2.3 cm in the second part of the duodenum. Vascular component of the lesion was better appreciated on endoscopic ultrasound color Doppler (Figure 1(a)). No lesions were identified in the esophagus or stomach. Exploratory laparotomy with duodenotomy and local lesion excision was planned. The lesion was excised with no intraoperative complications. 

Histologically, the tumor was identified in the submucosa and was composed of epithelioid cell nests, areas of spindle cells, and scattered ganglion cells. A positive cellular reaction for synaptophysin and S-100 was demonstrated by immunohistochemistry. The tumor was revealed to be a gangliocytic paraganglioma, an unusual lesion. Patient is on regular followup and is symptomatically better with no fresh complaints (Figures 1(b), 1(c), 1(d), 1(e), and 1(f)). 

## 3. Discussion

Gangliocytic paraganglioma of the duodenum was first reported by Dahl et al. in 1957. Kepes and Zacharias described the characteristics of light microscopy and electron microscopy findings [2]. Gangliocytic paraganglioma of the duodenum is extremely rare. In WHO classification of tumors of digestive tract (2000), gangliocytic paraganglioma was independently classified as a type of epithelial tumors. Other duodenal neuroendocrine tumors, except for undifferentiated neuroendocrine carcinoma, were classified as carcinoid tumors. Upper gastrointestinal bleeding is the main symptom of gangliocytic paraganglioma of the duodenum; however, the symptoms can appear as abdominal pain or abdominal discomfort [3]. Our patient presented with similar complaints. Males are affected slightly more commonly than females (1 to 1.8/1), and in terms of age at onset although the fifties are preferred, it has been encountered over an age range from 23 to 83 years [4, 5]. The present case was a 32-year-old male. 

The endoscopic features of gangliocytic paraganglioma do not differ from those of other submucosal tumors. Tumors can appear as polyps or lumps endoscopically, and biopsy results are usually negative because the tumors are submucosal. The majority of the reported duodenal gangliocytic paragangliomas is of benign and nonfunctional nature; therefore, radical surgery or lymph node dissection could be avoided with disease confirmation. Local surgical excision of the lesion is preferred. Some authors still emphasize that tumors of the duodenum often require pancreaticoduodenectomy or lymph node dissection. However, because metastasis and the recurrence of gangliocytic paraganglioma are rare and, moreover, no case of death resulting from this tumor has been reported, mass excision is considered sufficient to treat as long as abnormal features are not found in lymph nodes and bileand pancreatic ducts by endoscopic ultrasonography [4–6]. Gangliocytic paragangliomas are typically composed of, as in our case, epithelioid cell nests, areas of spindle cells, and scattered ganglion cells [6]. Immunohistochemically, chromogranin, synaptophysin, and S-100 are useful markers for confirmation of the lesion. The present tumor was immunocytochemically characterized by S-100 reactivity of the spindle cells and synaptophysin positive in other areas. Several authors have reported that epithelioid and ganglion cells are positive to neuroendocrine peptides, such as, somatostatin, pancreatic polypeptide, and serotonin. Continuous followup at the outpatient department for early detecting of recurrence is deemed necessary [4, 7, 8]. The present case underwent local excision and is on regular followup in the outpatient department.

## References

[1] Cohen T, Zweig SJ, Tallis A (1981). Paraganglioneuroma of the duodenum. Report of a case with radiographic findings, angiographic findings and a review of the literature. *American Journal of Gastroenterology*.

[2] Dahl EV, Waugh JM, Dahlin DC (1957). Gastrointestinalganglioneuromas, brief review with report of a duodenal ganglioneuroma. *American Journal of Pathology*.

[3] Kepes JJ, Zacharias DL (1971). Gangliocytic paragangliomas of the duodenum. A report of two cases with light and electron microscopic examination. *Cancer*.

[4] Hashimoto S, Kawasaki S, Matsuzawa K, Harada H, Makuuchi M (1992). Gangliocytic paraganglioma of the papilla of vater with regional lymph node metastasis. *American Journal of Gastroenterology*.

[5] Nagai T, Torishima R, Nakashima H (2004). Duodenal gangliocytic paraganglioma treated with endoscopic hemostasis and resection. *Journal of Gastroenterology*.

[6] Cooney T, Sweeney EC (1978). Paraganglioneuroma of the duodenum: an evolutionary hybrid?. *Journal of Clinical Pathology*.

[7] Wu G-C, Wang K-L, Zhang Z-T (2012). Gangliocytic paraganglioma of the duodenum: a case report. *Chinese Medical Journal*.

[8] Kwon J, Lee SE, Kang MJ, Jang J-Y, Kim S-W (2010). A case of gangliocytic paraganglioma in the ampulla of Vater. *World Journal of Surgical Oncology*.

